# Epidemiological analysis of pneumococcal strains isolated at Yangon Children’s Hospital in Myanmar via whole-genome sequencing-based methods

**DOI:** 10.1099/mgen.0.000523

**Published:** 2021-02-10

**Authors:** Masaya Yamaguchi, Hpoo Pwint Myo Win, Kotaro Higashi, Masayuki Ono, Yujiro Hirose, Daisuke Motooka, Daisuke Okuzaki, Mya Mya Aye, Moh Moh Htun, Hlaing Myat Thu, Shigetada Kawabata

**Affiliations:** ^1^​ Department of Oral and Molecular Microbiology, Osaka University Graduate School of Dentistry, Osaka, Japan; ^2^​ Bacteriology Research Division, Department of Medical Research, Ministry of Health and Sports, Yangon, Myanmar; ^3^​ Genome Information Research Center, Research Institute for Microbial Diseases, Osaka University, Suita, Osaka, Japan

**Keywords:** molecular epidemiology, Myanmar, pangenome, sequencing-based analysis, *Streptococcus pneumoniae*

## Abstract

*
Streptococcus pneumoniae* causes over one million deaths from lower respiratory infections per annum worldwide. Although mortality is very high in Southeast Asian countries, molecular epidemiological information remains unavailable for some countries. In this study, we report, for the first time, the whole-genome sequences and genetic profiles of pneumococcal strains isolated in Myanmar. We isolated 60 streptococcal strains from 300 children with acute respiratory infection at Yangon Children’s Hospital in Myanmar. We obtained whole-genome sequences and identified the species, serotypes, sequence types, antimicrobial resistance (AMR) profiles, virulence factor profiles and pangenome structure using sequencing-based analysis. Average nucleotide identity analysis indicated that 58 strains were *S. pneumoniae* and the other 2 strains were *Streptococcus mitis *. The major serotype was 19F (11 strains), followed by 6E (6B genetic variant; 7 strains) and 15 other serotypes; 5 untypable strains were also detected. Multilocus sequence typing analysis revealed 39 different sequence types, including 11 novel ones. In addition, genetic profiling indicated that AMR genes and mutations spread among pneumococcal strains in Myanmar. A minimum inhibitory concentration assay indicated that several pneumococcal strains had acquired azithromycin and tetracycline resistance, whereas no strains were found to be resistant against levofloxacin and high-dose penicillin G. Phylogenetic and pangenome analysis showed various pneumococcal lineages and that the pneumococcal strains contain a rich and mobile gene pool, providing them with the ability to adapt to selective pressures. This molecular epidemiological information can help in tracking global infection and supporting AMR control in addition to public health interventions in Myanmar.

## Data Summary

Data for the 60 sequenced streptococcal genomes were deposited into the DNA Data Bank of Japan (DDBJ) Sequence Read Archive (DRA), under the accession number DRA010197. The authors confirm all supporting data, code and protocols have been provided within the article or through supplementary data files.

**Impact Statement**

*
Streptococcus pneumoniae* causes lower respiratory infections with a high mortality rate, especially in children under 5 years old. Antibiotic selective pressure has recently been causing resistant pneumococcal clones to emerge and expand all over the world. In this study, we identified and analysed the genome sequences of 58 *S *. *pneumoniae* and 2 *S. mitis* strains isolated at Yangon Children’s Hospital in Myanmar, in Southeast Asia. This is the first report of pneumococcal genome sequences and profiles isolated in the country. Since the precise identification of bacterial species within the mitis group is not easy, we performed whole-genome average nucleotide identity analysis with representative bacterial genome sequences. In addition, we elucidated the distribution of capsular serotypes, sequence types (including 11 novel ones), antimicrobial resistance (AMR) genes and virulence factors, and also the pangenome structure. Phylogenetic and pangenome analyses suggested the presence of many novel *S. pneumoniae* lineages in Myanmar. Whole-genome sequence-based approaches can predict almost all serotypes with high accuracy in addition to species identification, sequence types and the distribution of AMR and virulence genes. This molecular epidemiological information would help in tracking global infection and supporting AMR control in addition to public health interventions in Myanmar.

## Introduction

*Streptococcus pneumoniae* is one of the major causes of bacterial pneumonia, meningitis and sepsis [1]. In 2016, this bacterium was estimated to be responsible for the deaths of approximately 1 190 000 people owing to lower respiratory infections all over the world [2]. Pneumococcal pneumonia is considered to be the most common cause of mortality in children younger than 5 years old [2]. *S. pneumoniae* has high natural genetic transformational ability and is able to acquire genes via horizontal gene transfer from closely related species, such as the *Streptococcus mitis* group. This genetic transformation confers antimicrobial resistance (AMR) to *S. pneumoniae* by genetic recombination of penicillin-binding proteins among related species, importing AMR genes, among others [3]. Currently, a 13-valent pneumococcal conjugated vaccine (PCV-13) and a 23-valent pneumococcal polysaccharide vaccine (PPV23) have been licensed and introduced in many countries. Because of the poor immunogenicity of PPV23 in infants, and its capsular polysaccharide vaccine-induced T cell-independent immune responses, the PCV-13 vaccine is generally administered to children [4]. These pneumococcal vaccines are a useful tool against AMR and have substantially reduced the global burden of pneumococcal infections [1]. However, they also lead to selective pressure on the covered serotypes, and thus non-vaccine serotypes of *S. pneumoniae* are increasing worldwide [5–7]. Thus, to control this infectious disease globally, it is important to collect and analyse molecular epidemiological information.

The Global Burden of Diseases, Injuries and Risk Factors (GBD) 2015 study estimated that the lower respiratory tract infection (LRI)-associated mortality in children under 5 years old in Myanmar was 164.3 children per 100 000 (95 % uncertainty interval: 115.6–238.9). They also estimated that pneumococcal pneumonia accounted for 61.2 % of LRI deaths among Southeast Asian children (younger than 5 years old) in 2015 [8]. The Myanmar government introduced a 10-valent pneumococcal conjugate vaccine (PCV-10) into the national immunization programme in 2016 and shifted to PCV-13 in December of 2018 through the support of Global Alliance for Vaccines and Immunization and United Nations Children’s Fund. Regarding the emergence and spread of AMR, Southeast Asia is viewed as high-risk region, since the inappropriate use of antibiotics is rampant and the implementation of policies to reduce these practices remains under development [9, 10]. *S. pneumoniae* is a pathogen with high levels of AMR; however, the detailed characteristics of *S. pneumoniae* isolated in Myanmar have remained unclear.

In this study, we collected swab samples isolated from 300 children with acute respiratory infection at Yangon Children’s Hospital, the largest children’s hospital in Myanmar. After microbiological testing, we isolated α-haemolytic Gram-positive *Streptococcus* from 60 samples. We performed whole-genome sequencing (WGS) analysis and identified the species, serotypes, sequence types (STs), AMR profiles, virulence factor profiles and pangenome structure via sequencing-based analysis.

## Methods

### Sample collection

We collected nasopharyngeal swab samples from inpatient children under 5 years of age who had been diagnosed with acute respiratory infection in Yangon Children’s Hospital during 2017–2019. Sampling was performed with written informed consent from the patients’ guardians, according to a protocol approved by the Institutional Review Board at the Department of Medical Research, Ministry of Health and Sports, Myanmar (Ethics/DMR/2017/083), and Osaka University Graduate School of Dentistry (H28-E26). All the swabs were streaked on trypticase soy (TS; BD Biosciences) or brain heart infusion (BHI; BD Biosciences) broth agar, with 5 % sheep blood, and incubated at 37 °C in an anaerobic jar with Anaero Pack (Mitsubishi Gas Chemical). Single colonies with α-haemolysis were picked up and the morphology was confirmed by microscopic observation with Gram staining. Isolated strains were stored in TS or BHI broth with 30 % glycerol at −80 °C.

### Next-generation sequencing

To extract bacterial genomic DNA, isolated strains were grown to mid-log phase in TS or BHI broth. Genomic DNA extraction was performed using the DNeasy PowerSoil kit (Qiagen) according to the manufacturer’s instructions. Purified DNA was quantified using the Qubit dsDNA HS Assay kit (Thermo Fisher Scientific). DNA degradation was evaluated by 1 % agarose gel electrophoresis using an E-Gel Electrophoresis System (Thermo Fisher Scientific). Genomic DNA was sheared using the Covaris S220 ultrasonicator (Covaris) and a library was prepared using the Nextera DNA Flex Library Prep Kit (Illumina). Paired-end sequencing (251 bp) was performed using the HiSeq 2500 or MiSeq platforms (Illumina). Data for the 60 sequenced streptococcal genomes were deposited into the DNA Data Bank of Japan (DDBJ) Sequence Read Archive (DRA), under the accession number DRA010197.

### Sequencing-based profiling analysis

**Fig. 1.**
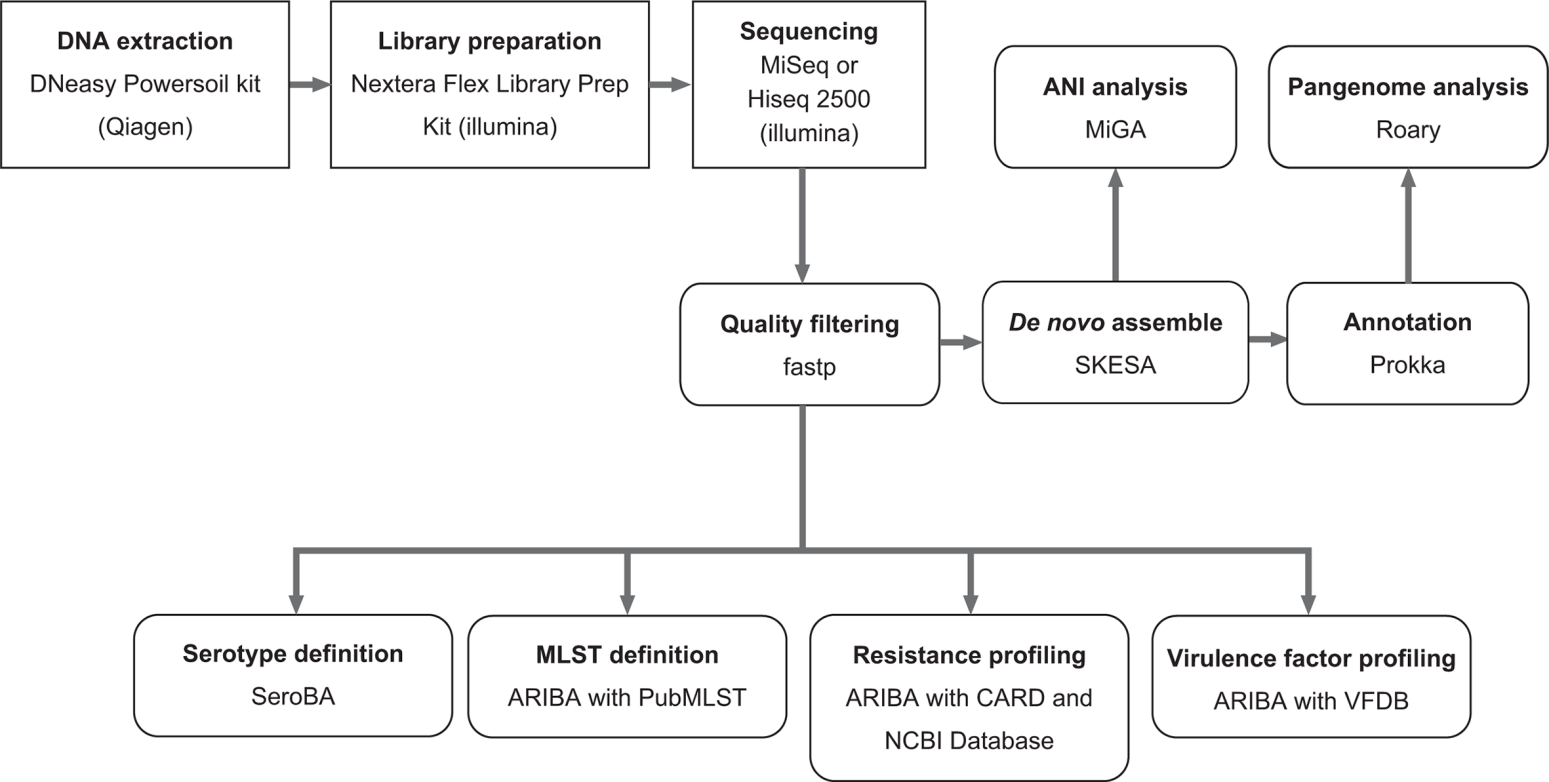
Workflow for sequencing-based methods to study pneumococcal strains. Rectangles indicate *in vitro* analysis, whereas rounded rectangles indicate *in silico* analysis.

The workflow is shown in Fig. 1. Quality control and preprocessing of the FASTQ files from the next-generation sequencing were performed using fastp v.0.20.0 [11]. *De novo* assembly was performed using the cleaned sequencing data and SKESA v.2.3.0 [12]. The assemblies were annotated with Prokka v.1.14.5 [13]. To identify the bacterial species, average nucleotide identity (ANI) analysis for the assemblies was performed using the Microbial Genomes Atlas webserver (http://microbial-genomes.org/) [14]. Pneumococcal capsular serotypes were identified using the cleaned sequencing data and SeroBA v.1.0.1 [15]. The ST, AMR and virulence factor profiles were determined using ARIBA 2.14.4 and the cleaned sequencing data [16]. We used the PubMLST (multilocus sequence typing) [17], the Comprehensive Antibiotic Resistance Database (CARD) v.3.0.8 [18], the National Center for Biotechnology Information (NCBI) Bacterial Antimicrobial Resistance Reference Gene Database (https://www.ncbi.nlm.nih.gov/bioproject/?term=PRJNA313047) and the core and full datasets of the virulence factor database (VFDB) [19] as reference databases for ST, AMR and virulence factor profiling, respectively. The minimum percentage identities for the assemblies were set to 93 and 90 for CARD and the other databases, respectively. The analysed data were visualized using Phandango [20].

### Minimum inhibitory concentration assay

A minimum inhibitory concentration (MIC) assay was conducted in accordance with the Clinical Laboratory Standards Institute (CLSI) guidelines as described previously with minor modifications [21–24]. *S. pneumoniae* strains were cultured at 37 °C in Todd–Hewitt broth (BD Biosciences) supplemented with 0.2 % yeast extract (THY; BD Biosciences). For the MIC assay, overnight pneumococcal culture was diluted 1 : 10 with fresh THY and grown to logarithmic phase (OD_600_ of 0.4–0.5). The bacterial culture was then washed and diluted 1 : 100 with phosphate-buffered saline (PBS). Bacteria (5 µl) were added to the individual wells of a 96-well plate containing 195 µl of Mueller–Hinton broth with lysed horse blood (Kyokuto Pharmaceutical Industrial) supplemented with twofold serial dilutions of antimicrobials, penicillin G (0.125–8 µg ml^−1^), azithromycin (0.031–2 µg ml^−1^), tetracycline (0.063–4 µg ml^−1^), chloramphenicol (0.125–8 µg ml^−1^), levofloxacin (0.125–8 µg ml^−1^), or clindamycin (0.016–1 µg ml^−1^). All antimicrobials were purchased from Tokyo Chemical Industry. Bacterial growth after 24 h at 37 °C in anaerobic conditions was measured spectrophotometrically at a wavelength of 600 nm. We defined OD_600_ values of <0.06 as complete inhibition of bacterial growth.

### Pangenome analysis

Pangenome analysis was performed using the Prokka-annotated pneumococcal genome sequences and Roary v.3.13.0 [25]. Using blastp with 95 % sequence identity, we clustered and performed an all-against-all comparison on the intact coding sequences. The genes were classified as core (99 % ≦ strains ≦ 100 %), soft core (95 % ≦ strains <99 %), shell (15 % ≦ strains <95 %), or cloud (0 % <strains <15 %) genes. In addition, total, conserved, unique and new genes were also calculated. ‘Total genes’ refers to the genes in the core and accessory among the genomes; ‘conserved genes’ refers solely to genes in the core; ‘unique genes’ refers to genes only present in one genome among the population; ‘new genes’ refers to genes in an added genome not present in the genomes that have been observed. Gene distribution was visualized using R v.3.6.3 in RStudio v.1.1.463 (https://rstudio.com/products/rstudio/). Core genome phylogenetic analysis was performed as described previously [21, 26–29], with minor modifications. Public pneumococcal genome information was obtained from the NCBI (https://www.ncbi.nlm.nih.gov/genome/browse/#!/prokaryotes/176/). The capsule types, MLSTs and accession numbers are listed in Table S1 (available in the online version of this articl). Briefly, single-nucleotide polymorphisms (SNPs) were extracted from aligned core genome sequences using SNP-sites v.2.5.1 [30]. The best-fitting codon evolutionary models for phylogenetic analyses were determined using Kakusan4 [31]. Markov chain Monte Carlo-based Bayesian data analysis was performed using MrBayes v.3.2.6 [32]. Samplings were performed until the standard deviation of split frequencies was 8×10^6^ generations. To validate phylogenetic inferences, maximum-likelihood phylogenetic trees with bootstrap values were generated using RAxML v.8.1.20 [33]. Phylogenetic trees were visualized using FigTree v.1.4.4 (https://github.com/rambaut/figtree) or iTOL [34].

## Results

### Species identification, serotyping and ST distribution

Between 2017–2019, we obtained 60 α-haemolytic streptococcal strains from 300 nasopharyngeal swab samples taken from inpatient children <5 years of age (Yangon Children’s Hospital) who had been diagnosed with acute respiratory infection. Among the 60 children from whom *Streptococcus* had been isolated, 29 had been fully vaccinated with PCV-10 (3 times), 3 had been partially vaccinated (once or twice), 27 had not been vaccinated and 1 had an unknown vaccination status, since the guardian could not provide a vaccination card (Table S2). Next-generation sequencing and *de novo* assembly were performed to construct the draft whole-genome sequences of these 60 strains. To identify the species of the clinically isolated strains, we performed whole-genome ANI analysis with representative bacterial genome sequences. ANI analysis represents the nucleotide-level similarity of all orthologous genes shared between any two prokaryotic genomes and offers robust resolution between strains of the same or closely related species. The 95 % ANI value corresponds to 70 % DNA–DNA hybridization relatedness, the traditional microbiological criterion to delineate bacterial species [35]. As a result, 58 strains showed a 98–99% ANI value for *S. pneumoniae* and the other 2 strains showed a ~95 % ANI value for *S. mitis* (Table S3).

**Fig. 2.**
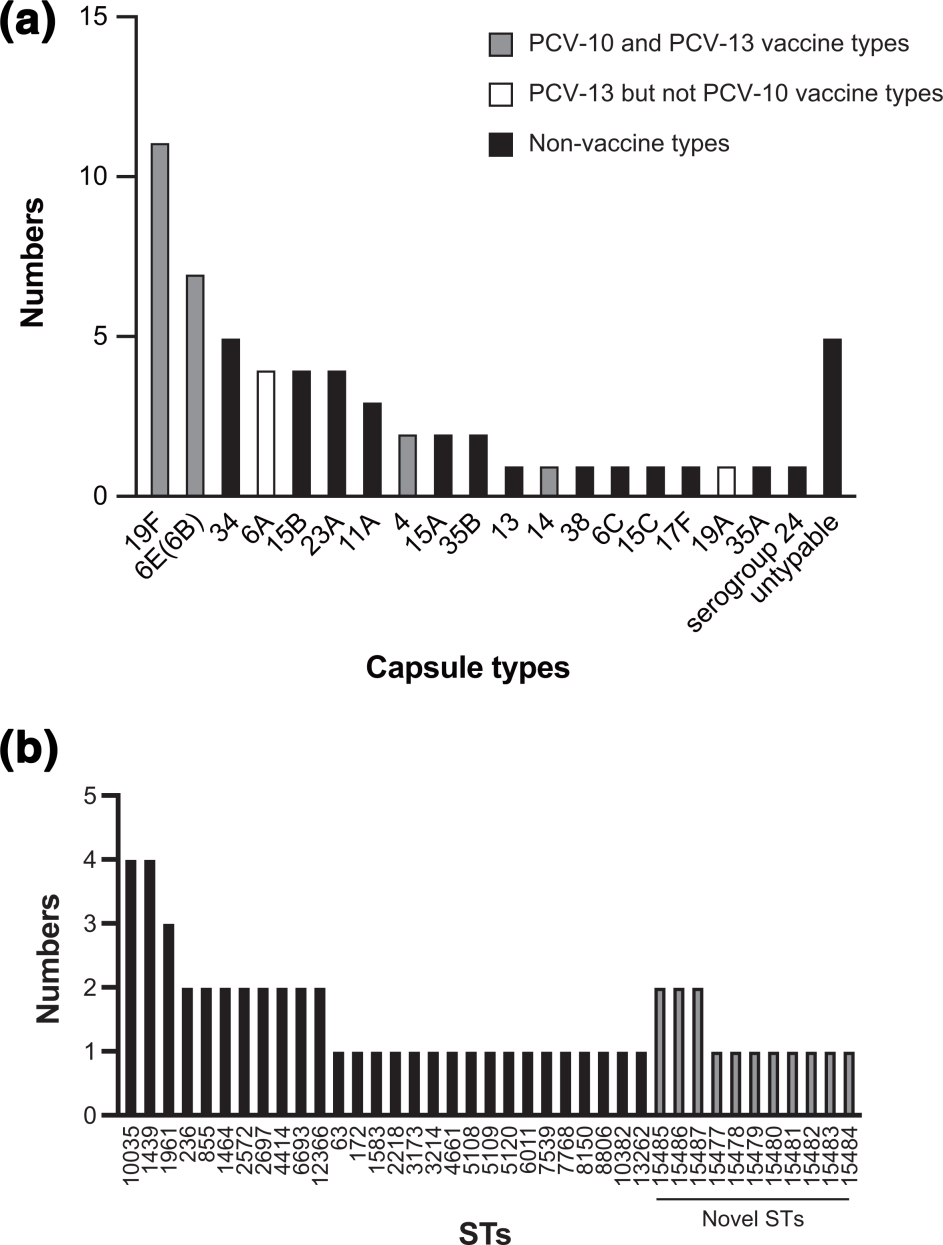
The distribution of the serotypes (a) and sequence types (STs) (b) in pneumococcal strains isolated in Yangon Children’s Hospital, Myanmar. Serotypes and STs were determined using the SeroBA and ARIBA programs with the PubMLST database, respectively. The correspondence between strains, serotypes and STs is shown in Table S3.

Next, we conducted *S. pneumoniae* serotyping using the WGS data. This sequencing-based serotyping showed that serotype 19F was dominant among these strains, and that 35 % (or 21 strains) and 43.3 % (or 26 strains) were PCV-10- and PCV-13-covered serotypes, respectively (Fig. 2a, Table S2). Serotype 6E is a genetic variant of serotype 6B and produces the 6B capsular polysaccharide [36]. Thus, we classified serotype 6E as a PCV-covered serotype. Ten PCV-10-covered strains were isolated from fully vaccinated children, and two serotype 4 strains were isolated from partially vaccinated children. The five untypable strains lacked *cps* genes in their genomes, indicating that the strains were acapsular. Meanwhile, we identified 39 different STs, with there being a small number of each (Table S3, Fig. 2b). Although ~5000 alleles and more than 15 000 STs of *S. pneumoniae* have been registered [17], we identified 4 novel alleles and 11 novel STs (Table S4). These results indicate the presence of various pneumococcal lineages in Yangon, Myanmar, including natively evolved ones.

We showed registered strain numbers by MLST and country in the PubMLST database on 5 December 2020 (Table S5). In the database, ST4414, ST5108, ST5109, ST5120, ST6693, ST10035 and ST10382 were strains that had been isolated from Thailand. In addition, ST1961 had been isolated from Thailand or Vietnam, ST8150 from Thailand or Nepal, and ST7539 from Nepal; these STs would be specific to Myanmar-neighbouring countries. Conversely, ST63, ST172, ST236, ST1439, ST1583, ST2218, ST2572, ST3214, ST4661 and ST8806 are global STs, isolated from multiple continents, including Southeast Asia. Certain STs have not been reported in other Southeast Asian countries. ST855 and ST1464 are also global STs, but have not been isolated from countries in Southeast Asia. ST2697 was isolated from the Republic of Korea or Russia, while ST3173 was isolated from the Republic of Korea or PR China. ST6011 and ST7768 were isolated from PR China, ST12366 from Turkey and ST13262 from the USA. These STs may enter Yangon from each country directly. The novel strains ST15477–15487 were only isolated this study.

### AMR and the distribution of virulence factors

**Fig. 3.**
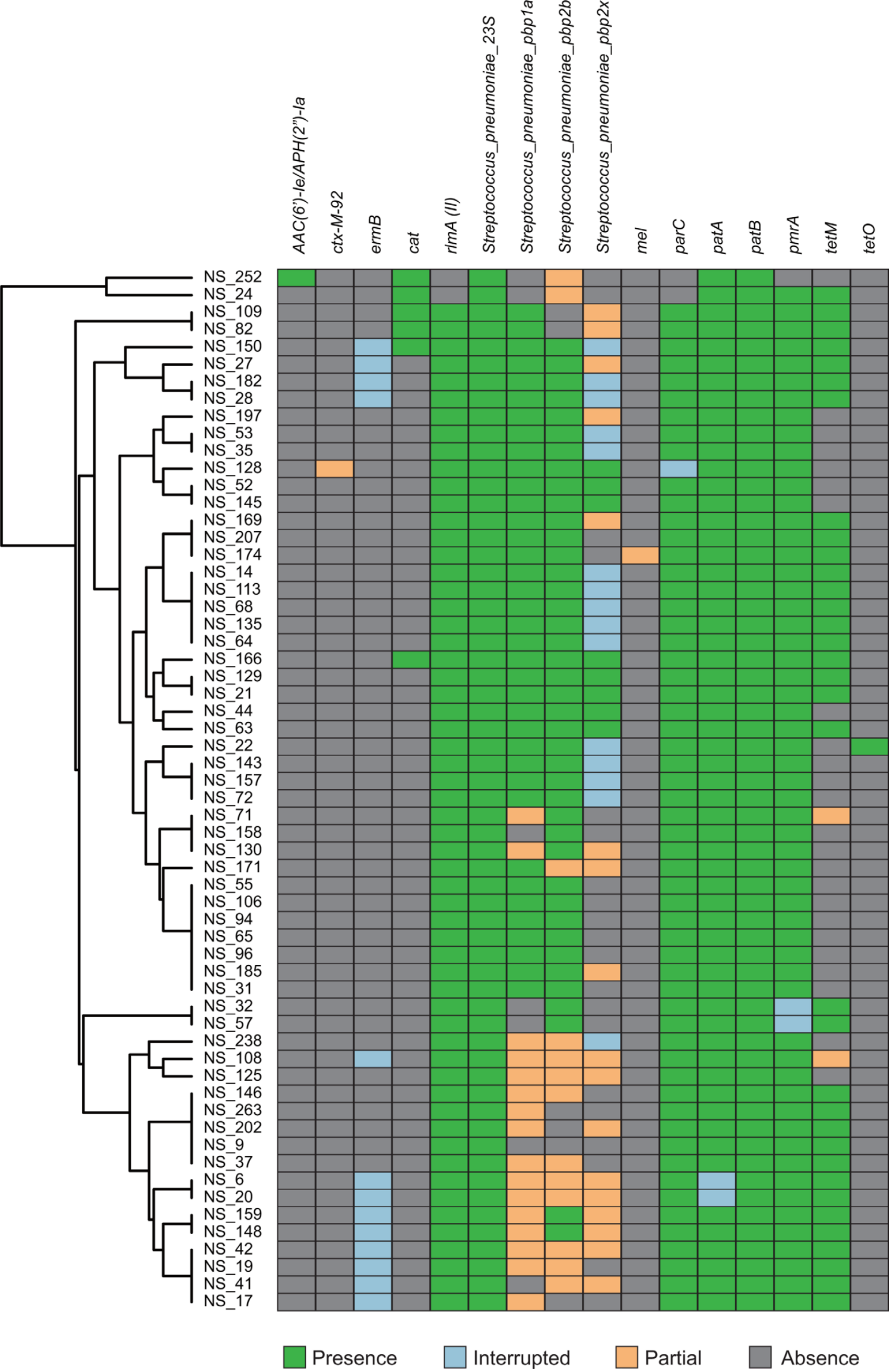
Burden of antimicrobial resistance (AMR) genes in the clinical strains including *Streptococcus mitis *. The reference data were obtained from the Comprehensive Antibiotic Resistance Database (CARD). Green, light blue, blue, orange and grey indicate matches to reference, interrupted, fragmented, partial and lacking genes, respectively. ‘Interrupted’ refers to an incomplete gene, lacking start and/or stop codons, ‘fragmented’ refers to a ene assembled to ≥2 contigs and ‘partial’ refers to not all of the reference being represented in the assembly. The clustering tree was generated by ARIBA based on the gene distribution. Graphical data were obtained using Phandango. The genes 23S rRNA, *pbp1a*, *pbp2b*, *pbp2x* and *parC* were determined to contain known variants contributing to AMR.

*S. pneumoniae* imports AMR genes by horizontal gene transfer. To identify the distribution and to find out which of the examined strains carry the various AMR genes among the clinical strains, we performed sequencing-based analysis (Figs 3 and S1). Regarding the mechanisms of penicillin resistance, we found mutations in the genes encoding penicillin-binding proteins (*pbp1a*, *pbp2x* and *pbp2b*), which can cause modifications in these proteins [18]. Conversely, we found no intact genes encoding β-lactamase. Concerning aminoglycoside and macrolide resistance, we were able to identify a gene encoding aminoglycoside acetyltransferase (*AAC(6′)-Ie*) and the genes *ermB*, *rlmA(II*), *mefA* and *msrD,* as well as mutations in the 23S rRNA. The *ermB* and *rlmA(II*) genes encode different 23S rRNA methyltransferases, whereas the *mefA* and *msrD* genes encode major facilitator superfamily (MFS)-type efflux proteins [18]. In addition, the chloramphenicol acetyltransferase is responsible for chloramphenicol resistance and the *tetM* and *tetO* genes encoding ribosomal protection proteins confer tetracycline resistance, respectively [18]. Almost all strains were determined to be carrying genes involved in quinolone resistance, *patA*, *patB* and *pmrA,* and mutations in *parC*. The *patA* and *patB* genes encode a heterodimeric ABC efflux pump, whereas the *pmrA* gene encodes an MFS-type efflux pump [18]. These results suggest the spread of the AMR genes, and that most pneumococcal strains in Myanmar are likely to exhibit AMR to multiple antibiotics.

We also examined the MICs of major antibiotics against pneumococcal infection, namely penicillin G, azithromycin, tetracycline, chloramphenicol, levofloxacin and clindamycin (Table 1). We used CLSI breakpoints to determine whether the strain was resistant, intermediate, or sensitive [24]. All 58 pneumococcal strains were susceptible to penicillin G, while the MICs for 2 *S *. *mitis* strains showed them to be resistant. The resistant strains did not vary greatly from known resistant gene profiles as compared to other pneumococcal strains (Figs 3 and S1), suggesting the presence of novel genes or mutations associated with penicillin resistance. There were 23 and 32 resistant strains to azithromycin and tetracycline, respectively, while a large minimal discrepancy was not found between resistant phenotypes and genes. In addition, all nine chloramphenicol-resistant strains contained the chloramphenicol acetyltransferase gene, while other susceptible strains did not. Although most strains carried quinolone resistance genes, only two *S. mitis* strains showed intermediate resistance, and there were no resistant strains. Finally, all 10 clindamycin-resistant strains, and 2 susceptible strains, carried the *ermB* gene. These results indicate that levofloxacin and high-dose penicillin G remain effective, whereas the genes and/or mutations involved in AMR are spreading.

**Table 1.** Minimum inhibitory concentrations (MICs) of the streptococcal strains in this study for penicillin G, azithromycin, tetracycline, chloramphenicol, levofloxacin and clindamycin

Strain	MIC (µg ml^−1^)					
	Penicillin G	Azithromycin	Tetracycline	Chloramphenicol	Levofloxacin	Clindamycin
NS_6	0.25	>2 (R)	4 (R)	4	1	>1 (R)
NS_9	1	>2 (R)	1	>8 (R)	1	0.125
NS_14	≤0.125	0.5	>4 (R)	2	0.5	0.063
NS_17	0.5	>2 (R)	>4 (R)	2	1	0.25
NS_19	0.5	>2 (R)	>4 (R)	2	1	0.25
NS_20	0.25	>2 (R)	4 (R)	4	1	>1 (R)
NS_21	≤0.125	0.5	>4 (R)	4	1	0.125
NS_22	≤0.125	1 (I)	2 (I)	>8 (R)	1	0.25
NS_24	8 (R)	>2 (R)	>4 (R)	8 (R)	4 (I)	0.25
NS_27	≤0.125	>2 (R)	>4 (R)	4	1	>1 (R)
NS_28	≤0.125	>2 (R)	>4 (R)	2	2	1 (R)
NS_31	≤0.125	1 (I)	1	0.5	2	0.25
NS_32	1	>2 (R)	>4 (R)	2	1	0.125
NS_35	≤0.125	0.5	1	0.5	1	0.125
NS_37	≤0.125	0.25	>4 (R)	2	1	0.063
NS_41	2	>2 (R)	>4 (R)	4	1	>1 (R)
NS_42	1	>2 (R)	1	>8 (R)	1	>1 (R)
NS_44	≤0.125	0.5	0.5	≤0.125	1	0.125
NS_52	≤0.125	1 (I)	0.125	2	1	0.125
NS_53	≤0.125	1 (I)	2 (I)	0.5	1	0.25
NS_55	≤0.125	1 (I)	0.25	2	2	0.125
NS_57	2	>2 (R)	>4 (R)	2	1	0.25
NS_63	≤0.125	0.5	2 (I)	2	2	0.25
NS_64	≤0.125	0.5	2 (I)	2	2	0.25
NS_65	0.25	1 (I)	0.25	2	1	0.25
NS_68	≤0.125	1 (I)	>4 (R)	4	1	0.25
NS_71	1	>2 (R)	>4 (R)	2	1	0.25
NS_72	≤0.125	0.5	0.25	2	1	0.125
NS_82	≤0.125	1 (I)	>4 (R)	>8 (R)	1	0.125
NS_94	≤0.125	0.5	0.25	2	1	0.031
NS_96	≤0.125	1 (I)	0.25	2	2	0.125
NS_106	≤0.125	1 (I)	0.25	2	1	0.125
NS_108	2	>2 (R)	>4 (R)	2	2	>1 (R)
NS_109	0.5	0.5	>4 (R)	>8 (R)	1	0.125
NS_113	≤0.125	0.25	>4 (R)	2	1	0.063
NS_125	1	0.5	0.5	4	2	0.125
NS_128	≤0.125	1 (I)	0.5	2	1	0.125
NS_129	≤0.125	0.5	>4 (R)	2	1	0.125
NS_130	2	0.5	0.5	4	2	0.25
NS_135	≤0.125	0.5	>4 (R)	4	2	0.125
NS_143	≤0.125	0.5	0.25	4	1	0.125
NS_145	≤0.125	1 (I)	0.25	4	1	0.125
NS_146	1	>2 (R)	>4 (R)	2	1	0.25
NS_148	1	>2 (R)	>4 (R)	4	2	>1 (R)
NS_150	≤0.125	>2 (R)	>4 (R)	8 (R)	0.5	0.125
NS_157	≤0.125	1 (I)	0.25	4	1	0.125
NS_158	1	1 (I)	0.25	2	1	0.25
NS_159	1	>2 (R)	>4 (R)	4	1	>1 (R)
NS_166	≤0.125	1 (I)	>4 (R)	8 (R)	1	0.125
NS_169	≤0.125	1 (I)	>4 (R)	4	1	0.125
NS_171	≤0.125	0.5	0.25	4	1	0.063
NS_174	≤0.125	>2 (R)	>4 (R)	2	1	0.125
NS_182	≤0.125	>2 (R)	>4 (R)	4	1	>1 (R)
NS_185	≤0.125	0.5	0.25	4	1	0.125
NS_197	≤0.125	1 (I)	0.25	4	2	0.125
NS_202	2	>2 (R)	>4 (R)	4	1	0.25
NS_207	≤0.125	1 (I)	>4 (R)	2	1	0.125
NS_238	0.25	1 (I)	0.125	4	2	0.125
NS_252	>8 (R)	>2 (R)	0.5	8 (R)	4 (I)	0.125
NS_263	2	>2 (R)	>4 (R)	2	1	0.25

R, resistant; I, intermediate. The penicillin breakpoints were for patients without meningitis who are treated intravenously.

**Fig. 4.**
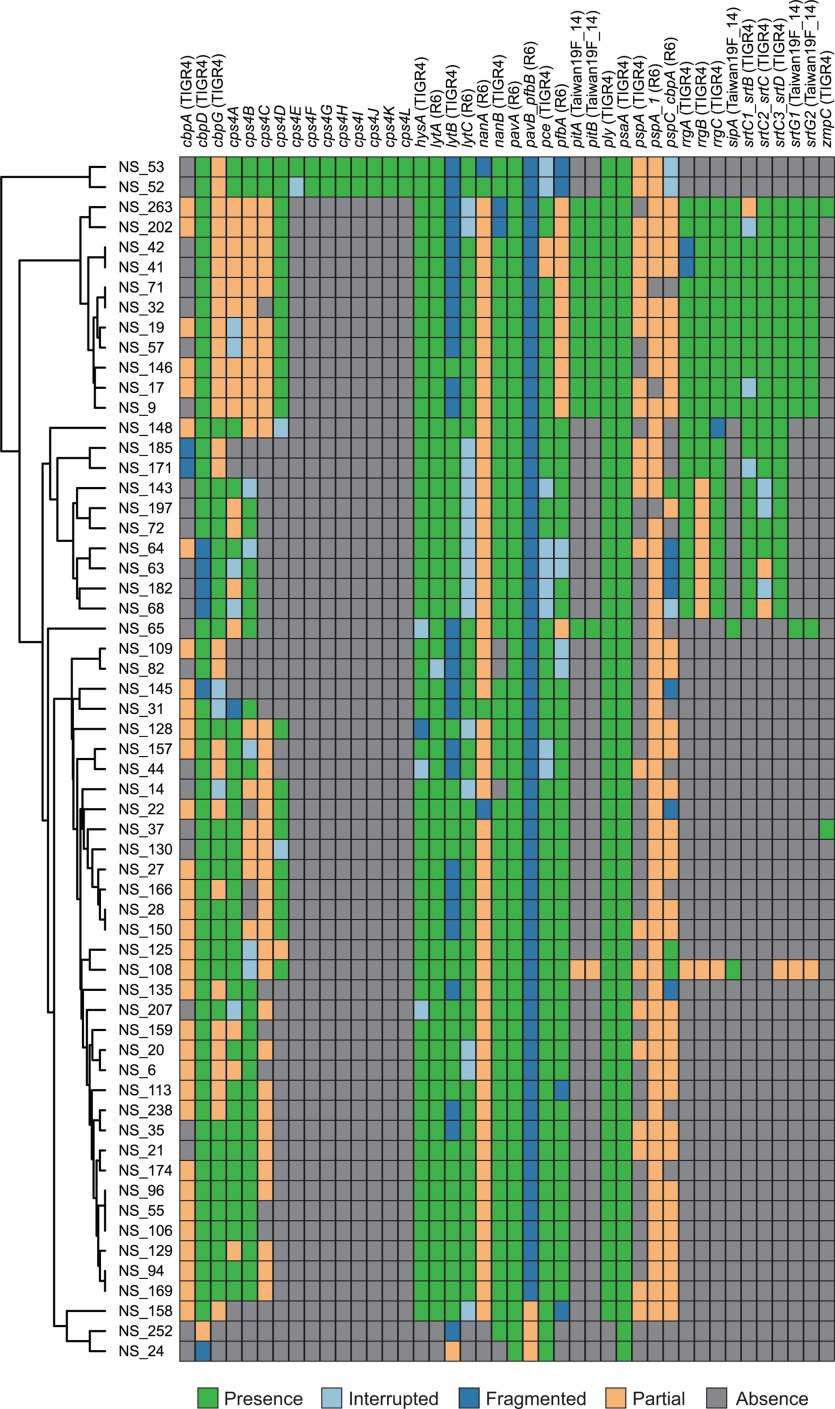
Burden of genes encoding virulence factors in the clinical strains including *Streptococcus mitis *. The reference data were obtained from virulence factor database (VFDB) core dataset. Green, light blue, blue, orange and grey indicate matches to reference, interrupted, fragmented, partial and lacking genes, respectively. The clustering tree was generated using ARIBA based on the gene distribution. Graphical data were obtained using Phandango.

Next, we detected pneumococcal virulence factors using VFDB [19]. Gene mapping using VFDB core and full databases indicated that *psaA*, *pavA*, *cppA*, *eno*, *htrA*, *plr*/*gapA* and *tig*/*ropA* are conserved as intact genes among all strains, including the two *S. mitis* strains (Figs 4 and S2). In addition, *cbpD*, *lmb*, *pavB/pfbB*, *pce* and *slrA* were present in all strains. On the other hand, the *ply*, *hysA/SpnHL*, *lytA*, *nanA*, *piaA* and *srtA* genes are conserved as intact genes, and the *cbpG*, *lytC*, *iga*, *pfbA* and *piuA* genes are present in 58 pneumococcal strains. Most of these genes encode pneumococcal cell surface proteins. The *cbpD*, *pce/cbpE*, *lytA* and *cbpG* genes encode choline-binding proteins, *hysA/SpnHL*, *nanA* and *pfbA* encode cell wall anchoring proteins, *psaA*, *slrA*, *piaA* and *piuA* encode lipoproteins, and *pavA*, *eno*, *plr*/*gapA* and *iga* encode non-classical proteins [21, 27, 37]. Our sequencing-based profiling revealed the distribution of virulence factors in addition to AMR genes among the clinical strains. This information is important for selecting pneumococcal vaccine candidates; however, the mapping method is not suitable for detecting genes containing sequence diversity, such as *cbpA*, *pspA* and many others. Therefore, to analyse the precise distribution of these genes, further phylogenetic analysis would be needed.

### Pangenome analysis of *S. pneumoniae* isolated in Yangon

**Fig. 5.**
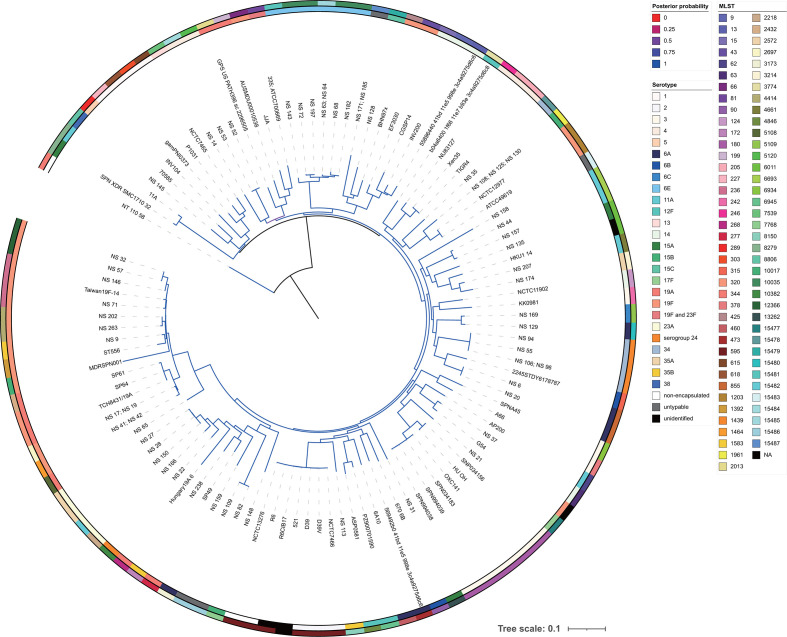
Bayesian phylogenetic relationship of 58 pneumococcal strains isolated from Yangon and 58 public whole-genome-sequenced strains. The phylogenetic tree was calculated by MrBayes using pneumococcal core gene single-nucleotide polymorphisms (SNPs). The tree is midpoint-rooted; the scale bar indicates nucleotide substitutions per site, and the colour gradient of the branches indicates posterior probability. Inner and outer circles show serotypes and MLSTs, respectively. Serotypes and MLSTs are shown as indicated in the figure. Only one node contains two different serotypes, 19F (strain 335) and 23F (strain ATCC 700669).

**Fig. 6.**
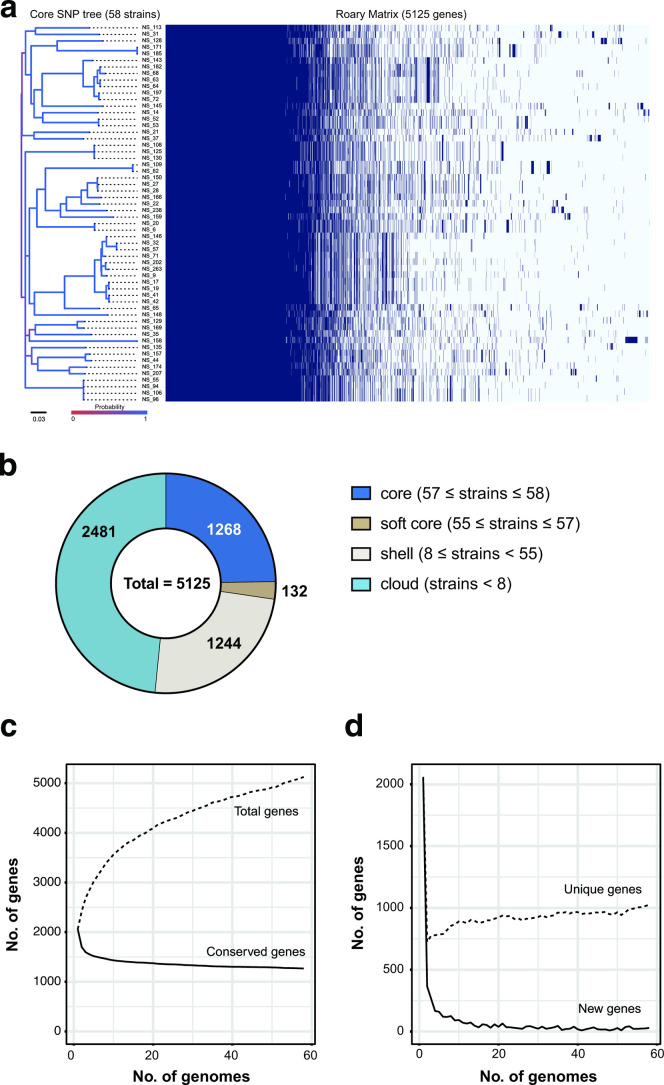
Pangenome analysis of the 58 pneumococcal strains. (a) Bayesian phylogenetic tree based on pneumococcal core gene single-nucleotide polymorphisms (SNPs) and Roary matrix. The tree is midpoint-rooted. The scale bar indicates nucleotide substitutions per site. The colour gradient of the phylogenetic tree indicates posterior probability. The Roary matrix shows whether individual genes are present in the strains. (b) Pangenome pie chart showing the number of core and accessory genes. Accessory genes were divided into soft core, shell and cloud genes. (c) Conserved and total gene numbers in the pangenome. (d) Novel and unique gene numbers in the pangenome. These graphs indicate how the pangenome varies as genomes are added.

We conducted Bayesian and maximum-likelihood phylogenetic analysis using 58 pneumococcal genomes from this study and 58 public ones. Both analyses produced almost identical phylogenetic trees (Figs 5 and S3). Pneumococcal strains from Yangon, Myanmar did not form a single region-specific cluster, but several small clusters related to global ones. In addition, some strains, namely NS63 (ST15485), NS64 (ST15485), NS68 (ST10035), NS143 (ST15481), NS182 (ST10035) and NS197 (ST10035), formed a small cluster without global strains. We also performed pangenome analysis on 58 pneumococcal strains. Roary analysis indicated that the 58 strains contained 5125 different genes. Out of these, 1268 (24.7 %) were classified as core genes, and 3857 (75.3 %) as accessory genes. Each strain contained 2000–2200 coding sequences. Interestingly, the MLST analysis revealed high genetic diversity among these strains (Fig. 6a, b), considering that they were isolated from one country (Myanmar). Pangenome analysis also showed that as the number of strains analysed increased, so did the number of unique genes. Moreover, the number of novel genes did not approach 0 and the total size of the pangenome did not stabilize (Fig. 6c, d). These results illustrated various pneumococcal lineages both related and not related to global strains, and indicated that some strains can evolve to adapt to a region-specific environment. Furthermore, these results imply that as a species, pneumococcal strains contain a rich, mobile gene pool.

## Discussion

In this study, we report for the first time the whole-genome sequences and genetic profiles of clinically isolated pneumococcal strains in Myanmar. Although the isolation area is limited to Yangon, the largest city in Myanmar, the distribution of pneumococcal serotypes and AMR genes is vital information for public health interventions in the country. Moreover, any information on the genetic sequences of pneumococcal virulence factors could aid investigations into novel vaccine antigen candidates. Furthermore, this molecular epidemiological information will support global infection and AMR control. Recently, various genetic and statistical strategies have helped researchers generate novel potential drug target candidates. Previously, we reported that molecular evolutionary analysis using bacterial whole-genome sequences could be a powerful tool for revealing the importance of virulence factors in tracking the infections and their transmissions [26–28]. Recently, a genome-wide association study using host and pneumococcal genome sequences identified host genetic variants and pathogen genes associated with susceptibility to pneumococcal meningitis [38]. In addition, a comparative genomics approach that used the global diversities of *S. pyogenes* strains enabled the targeting of immunogenic epitopes within antigens that were less amenable to variation [39]. The compilation of bacterial whole-genome sequences worldwide would contribute to the development and enhancement of recent global medical strategies.

The precise identification of bacterial species is not easy. Conventional phenotypic methods, such as 16S rRNA sequencing and mass spectrometry analysis, are often unable to distinguish the bacterial species within the mitis group streptococci [40, 41]. In addition, further analysis is often required to identify the capsular serotypes, STs, AMR abilities, etc. In particular, the epidemiology of pneumococcal capsular serotypes is essential for public health, as polysaccharide capsules are used as antigens in commercial vaccines. Pneumococcal serotyping requires specific monoclonal antibody or multiplex PCR primer sets (https://www.cdc.gov/streplab/pneumococcus/resources.html). Commercial pneumococcal serotyping kits and multiplex PCR methods are limited, as they can only identify ~40 of 100 serotypes [42]. In addition to species identification, STs, and the distribution of AMR and virulence genes, WGS-based approaches can predict almost all serotypes with high accuracy. Although both sequencing analysis and bioinformatic techniques currently have some limitations (e.g. cost and time), the rapid advances in these technologies will hopefully be able to solve these problems in the near future.

Our analysis showed that 35 and 43.3 % of the strains were PCV-10- and PCV-13-covered serotypes, respectively. Among the 29 strains isolated from fully vaccinated children, 10 were PCV-10-covered serotypes, while the remaining 19 strains were not. Five of those 10 strains were 19F, while the other 5 were 6E (6B). The effectiveness for 19F and 6E (6B) might have been lower than that for other serotypes in Myanmar. In addition, five strains were covered by PCV-13 but not PCV-10. This result indicates that the introduction of PCV-13 would be able to protect more children from pneumococcal infections in Myanmar. At the same time, the selective pressure from PCV-13 may decrease the total number of patients but could cause serotype replacement, as it did in other countries that introduced PCV-13.

The genes involved in AMR and virulence can spread among *S. pneumoniae *-related species by horizontal gene transfer [3, 43]. In other words, pneumococcal AMR genes and virulence factors can spread globally, and regular surveillance and monitoring are crucial. In Myanmar, as well as other Southeast Asian countries, easy access to antibiotics may lead to improper use and/or overuse, thus increasing the selective pressure on bacterial populations. We identified various mutations in multiple AMR genes, whereas β-lactamase genes conferring high β-lactam resistance were not detected. However, since mapping-based detection methods are highly dependent on genetic databases, they cannot detect novel mutations and genes contributing to AMR and/or virulence. In addition, it remains challenging to detect repeat sequences accurately using short-read data, which is a limitation. To investigate the role of novel genes, further molecular biological experiments are required.

Fortunately, there were no penicillin G- and/or levofloxacin-resistant pneumococcal strains, although there were numerous azithromycin- and/or tetracycline-resistant strains. Most pneumococcal strains carried mutations or genes associated with penicillin and quinolone resistance. Pneumococcal resistance to quinolones is induced by accumulated mutations in genes such as *gyrA*, *parC* and *parE*, increased efflux relating to *pmrA*, *patA* and *patB*, or acquisition of plasmids encoding Qnr proteins [44]. Improper use of levofloxacin may cause high-level *S. pneumoniae* resistance, since our pangenome analysis indicated that pneumococcal strains contain a rich and mobile gene pool. The presence of lineages related to global strains also suggests that AMR genes may be imported, and are transmitted globally. The combination of bacterial genomics and MIC assay suggested that vigilance and antimicrobial stewardship based on these results are required to maintain the efficacy of penicillin and respiratory fluoroquinolones against pneumococcal infections.

This is the first report of pneumococcal whole-genome sequences and their molecular epidemiological characterization from Myanmar. Whole-genome sequences are a valuable resource, as their phylogenetic relationships can reveal evolutionary directions and transmission routes. However, since sequencing-based approaches require easily accessible next-generation sequencers, large computational resources and regular maintenance [45], it is not easy to introduce this strategy in developing countries. In this study, researchers from Myanmar collected the specimens and performed conventional phenotyping methods, whereas Japanese researchers performed next-generation sequencing and bioinformatics analysis. This collaborative effort could thus be a model for other similar research endeavours that would aim to collect molecular epidemiological information from developing countries.

## Supplementary Data

Supplementary material 1

Supplementary material 2
