# Supplementary material for: Epidemiological analysis of pneumococcal strains isolated at Yangon Children’s Hospital in Myanmar via whole-genome sequencing-based methods
Source: Microb Genom. 2021 Feb 10;7(2):000523. doi: 10.1099/mgen.0.000523 (PMC8208701; doi:10.1099/mgen.0.000523)
Supplement: Supplementary material 1 [file mgen-7-523-s001.pdf]

**Supplementary Data**

# Epidemiological Analysis of Pneumococcal Strains Isolated at Yangon Children's Hospital in Myanmar via Whole-genome Sequencing-based Methods

Masaya Yamaguchi[a*], Hpoo Pwint Myo Win[b], Kotaro Higashi[a], Masayuki Ono[a], Yujiro Hirose[a], Daisuke Motooka[c], Daisuke Okuzaki[c], Mya Mya Aye[b], Moh Moh Htun[b], Hlaing Myat Thu[b], and Shigetada Kawabata[a*]

*[a]Department of Oral and Molecular Microbiology, Osaka University Graduate School of Dentistry, Osaka, Japan; [b]Bacteriologyearesearch Division, Department of Medical Research, Ministry of Health and Sports, Yangon, Myanmar; [c]Genome Information Research Center, Research Institute for Microbial Diseases, Osaka University, Suita, Osaka, Japan*

[*]Corresponding author:

Masaya Yamaguchi: yamaguchi@dent.osaka-u.ac.jp

Shigetada Kawabata: kawabata@dent.osaka-u.ac.jp

**Supplementary Table 1.** Summary of public pneumococcal genomes used in this study

| Strain | Species | Capsule type | ST | Accession No. |
|---|---|---|---|---|
| NCTC7465 | *S. pneumoniae* | 1 | 615 | LN831051.1 |
| NT_110_58 | *S. pneumoniae* | non-encapsulated | 344 | CP007593.1 |
| Hungary19A-6 | *S. pneumoniae* | 19A | 268 | CP000936.1 |
| 670-6B | *S. pneumoniae* | 6B | 90 | CP002176.1 |
| ATCC 700669 | *S. pneumoniae* | 23F | 81 | FM211187.1 |
| 335 | *S. pneumoniae* | 19F | 81 | CP026670.1 |
| 6A-10 | *S. pneumoniae* | 6A | 460 | CP053210.1 |
| CGSP14 | *S. pneumoniae* | 14 | 15 | CP001033.1 |
| SP49 | *S. pneumoniae* | 19A | 277 | CP018136.1 |
| 2245STDY6178787 | *S. pneumoniae* | 6B | 315 | LR216060.1 |
| 70585 | *S. pneumoniae* | 5 | 289 | CP000918.1 |
| NU83127 | *S. pneumoniae* | 4 | 246 | AP018936.1 |
| Xen35 | *S. pneumoniae* | 4 | 205 | CP025256.1 |
| TIGR4 | *S. pneumoniae* | 4 | 205 | AE005672.3 |
| ASP0581 | *S. pneumoniae* | 12F | 4846 | AP019192.2 |
| ST556 | *S. pneumoniae* | 19F | 1392 | CP003357.2 |
| KK0981 | *S. pneumoniae* | 3 | 242 | AP017971.1 |
| PZ900701590 | *S. pneumoniae* | 12F | 6945 | CP050175.1 |
| INV104 | *S. pneumoniae* | 1 | 227 | FQ312030.1 |
| AP200 | *S. pneumoniae* | 11A | 62 | CP002121.1 |
| A45 | *S. pneumoniae* | 3 | 6934 | HE983624.1 |
| BHN97x | *S. pneumoniae* | 19F | 425 | CP025076.1 |
| 55896440-41bd-11e5-998e-3c4a9275d6c6 | *S. pneumoniae* | 14 | 13 | LR216065.1 |
| JJA | *S. pneumoniae* | 19F | 66 | CP000919.1 |
| NCTC12977 | *S. pneumoniae* | 19F | 1203 | LR134294.1 |
| Taiwan19F-14 | *S. pneumoniae* | 19F | 236 | CP000921.1 |
| P1031 | *S. pneumoniae* | 1 | 303 | CP000920.1 |
| EF3030 | *S. pneumoniae* | 19F | 43 | CP035897.1 |
| ATCC 49619 | *S. pneumoniae* | 19F | 1203 | AP018938.1 |
| INV200 | *S. pneumoniae* | 14 | 9 | FQ312029.1 |
| NCTC11902 | *S. pneumoniae* | 14 | 124 | LS483417.1 |
| AUSMDU00010538 | *S. pneumoniae* | 19A | 199 | CP045931.1 |
| TCH8431/19A | *S. pneumoniae* | 19A | 320 | CP001993.1 |
| 11A | *S. pneumoniae* | 11A | 8279 | CP018838.1 |
| G54 | *S. pneumoniae* | 19F | NA* | CP001015.1 |
| GPS_US_PATH396-sc-2296505 | *S. pneumoniae* | 19A | 2013 | LR216050.1 |
| b04a6400-1f66-11e7-b93e-3c4a9275d6c8 | *S. pneumoniae* | 12F | 3774 | LR536843.1 |
| SP64 | *S. pneumoniae* | 19A | 320 | CP018138.1 |
| SP61 | *S. pneumoniae* | 19A | 2432 | CP018137.1 |
| 569492b0-41bd-11e5-998e-3c4a9275d6c6 | *S. pneumoniae* | 6A | 473 | LR216064.1 |
| gamPNI0373 | *S. pneumoniae* | 1 | 618 | CP001845.1 |
| HU-OH | *S. pneumoniae* | 3 | 180 | AP018937.1 |
| SPN XDR SMC1710-32 | *S. pneumoniae* | 15A | 8279 | CP025838.1 |
| NCTC7466 | *S. pneumoniae* | 2 | 595 | LS483374.1 |
| D39V | *S. pneumoniae* | 2 | 595 | CP027540.1 |
| D39 | *S. pneumoniae* | 2 | 595 | CP000410.2 |
| MDRSPN001 | *S. pneumoniae* | 19F | 10017 | AP018391.1 |
| NCTC13276 | *S. pneumoniae* | non-encapsulated | 595 | LS483390.1 |
| R6CIB17 | *S. pneumoniae* | unidentified | 595 | CP038808.1 |
| R6 | *S. pneumoniae* | non-encapsulated | 595 | AE007317.1 |
| HKU1-14 | *S. pneumoniae* | unidentified | 6011 | CP019299.1 |
| SNP034183 | *S. pneumoniae* | 3 | 180 | FQ312043.1 |
| OXC141 | *S. pneumoniae* | 3 | 180 | FQ312027.1 |
| SNP994039 | *S. pneumoniae* | 3 | 180 | FQ312044.2 |
| SNP994038 | *S. pneumoniae* | 3 | 180 | FQ312041.2 |
| SNP034156 | *S. pneumoniae* | 3 | 180 | FQ312045.1 |
| A66 | *S. pneumoniae* | 3 | 378 | LN847353.1 |
| 521 | *S. pneumoniae* | unidentified | NA* | CP036529.1 |

*NA means the strain contains only 6 of 7 MLST genes.

**Supplementary Table 2.** Patient information in this study

| Strain | Age | Sex | PCV-10 vaccination | Capsule type |
|---|---|---|---|---|
| NS_6 | 4 months | F | No | 06A |
| NS_9 | 8 months | M | Fully vaccinated | 19F |
| NS_14 | 7 months | F | Fully vaccinated | 13 |
| NS_17 | 1 year and 8 months | F | No | 19F |
| NS_19 | 2 years and 5 months | F | No | 19F |
| NS_20 | 10 months | F | Fully vaccinated | 06A |
| NS_21 | 1 year | M | Fully vaccinated | 17F |
| NS_22 | 3 months | M | No | serogroup 24 |
| NS_24 | 1 year and 5 months | M | Fully vaccinated | N/A |
| NS_27 | 1 year and 2 months | M | Fully vaccinated | 23A |
| NS_28 | 11 months | M | Fully vaccinated | 23A |
| NS_31 | 10 months | F | Fully vaccinated | 15A |
| NS_32 | 9 months | F | Fully vaccinated | 19F |
| NS_35 | 3 months | M | No | 34 |
| NS_37 | 2 months | M | No | 14 |
| NS_41 | 1 year | M | Unknown | 19F |
| NS_42 | 10 months | M | Fully vaccinated | 19F |
| NS_44 | 7 months | F | Fully vaccinated | 11A |
| NS_52 | 3 months | F | Vaccinated once in 2 months | 4 |
| NS_53 | 6 months | M | Vaccinated in 2 and 4 months | 4 |
| NS_55 | 9 months | M | Fully vaccinated | 34 |
| NS_57 | 8 months | M | Fully vaccinated | 19F |
| NS_63 | 8 months | M | Fully vaccinated | 6E(6B) |
| NS_64 | 8 months | M | Fully vaccinated | 6E(6B) |
| NS_65 | 8 months | M | Fully vaccinated | 19A |
| NS_68 | 1 year and 1 month | M | Fully vaccinated | 6E(6B) |
| NS_71 | 2 years and 2 months | M | No | 19F |
| NS_72 | 11 months | M | Fully vaccinated | 6E(6B) |
| NS_82 | 11 months | F | No | untypable |
| NS_94 | 1 year and 7 months | F | No | 34 |
| NS_96 | 1 year and 2 months | M | No | 34 |
| NS_106 | 1 year and 3 months | M | Fully vaccinated | 34 |
| NS_108 | 1 year and 7 months | M | No | 15B |
| NS_109 | 5 months | M | No | untypable |
| NS_113 | 8 months | F | Fully vaccinated | 35B |
| NS_125 | 1 year and 10 months | M | Fully vaccinated | 15B |
| NS_128 | 1 year and 9 months | M | No | 15C |
| NS_129 | 7 months | M | vaccinated at 2 and 4 months | 06A |
| NS_130 | 7 months | M | Fully vaccinated | 15B |
| NS_135 | 5 months | M | No | 15A |
| NS_143 | 7 months | F | No | 6E(6B) |
| NS_145 | 2 year | M | No | 38 |
| NS_146 | 1 year and 6 months | M | Fully vaccinated | 19F |
| NS_148 | 1 year and 7 months | M | No | 15B |
| NS_150 | 10 months | M | Fully vaccinated | 23A |
| NS_157 | 1 year and 9 months | F | No | 11A |
| NS_158 | 3 months | F | No | untypable |
| NS_159 | 1 year | F | Fully vaccinated | 06A |
| NS_166 | 3 years | M | No | 23A |
| NS_169 | 1 year and 3 months | M | Fully vaccinated | 06C |
| NS_171 | 3 years and 2 months | F | No | untypable |
| NS_174 | 8 months | M | Fully vaccinated | 35A |
| NS_182 | 2 months | M | No | 6E(6B) |
| NS_185 | 3 months | M | No | untypable |
| NS_197 | 1 year and 10 months | M | Fully vaccinated | 6E(6B) |
| NS_202 | 4 months | M | No | 19F |
| NS_207 | 6 months | M | No | 11A |
| NS_238 | 1 year and 6 months | M | No | 35B |
| NS_252 | 1 year | M | Fully vaccinated | N/A |
| NS_263 | 12 months | M | No | 19F |

**Supplementary Table 3.** Summary of the streptococcal clinical isolates in this study

| Strain | Species | Capsule type | ST | The closest relatives found by MiGA in the database | |
|---|---|---|---|---|---|
| NS_6 | *S. pneumoniae* | 06A | 855 | *S. pneumoniae* NZ LR216060 (98.99% ANI) | *S. pneumoniae* R6 NC 003098 (98.8% ANI) |
| NS_9 | *S. pneumoniae* | 19F | 1583 | *S. pneumoniae* A026 NC 022655 (99.74% ANI) | *S. pneumoniae* NZ AP018391 (99.23% ANI) |
| NS_14 | *S. pneumoniae* | 13 | 7539 | *S. pneumoniae* R6 NC 003098 (98.81% ANI) | *S. pneumoniae* NZ LR216037 (98.8% ANI) |
| NS_17 | *S. pneumoniae* | 19F | 2697 | *S. pneumoniae* A026 NC 022655 (99.91% ANI) | *S. pneumoniae* NZ AP018391 (99.16% ANI) |
| NS_19 | *S. pneumoniae* | 19F | 2697 | *S. pneumoniae* A026 NC 022655 (99.91% ANI) | *S. pneumoniae* NZ AP018391 (99.17% ANI) |
| NS_20 | *S. pneumoniae* | 06A | 855 | *S. pneumoniae* NZ LR216060 (99.01% ANI) | *S. pneumoniae* R6 NC 003098 (98.78% ANI) |
| NS_21 | *S. pneumoniae* | 17F | 15477* | *S. pneumoniae* R6 NC 003098 (98.87% ANI) | *S. pneumoniae* NZ LR216037 (98.86% ANI) |
| NS_22 | *S. pneumoniae* | serogroup 24 | 10382 | *S. pneumoniae* NZ CP028436 (99.06% ANI) | *S. pneumoniae* NZ LR536837 (99.03% ANI) |
| NS_24 | *S. mitis* | N/A | N/A | *S. mitis* NZ CP028415 (95.91% ANI) | *S. mitis* B6 NC 013853 (94.55% ANI) |
| NS_27 | *S. pneumoniae* | 23A | 2572 | *S. pneumoniae* NZ CP018136 (98.97% ANI) | *S. pneumoniae* NZ CP028436 (98.95% ANI) |
| NS_28 | *S. pneumoniae* | 23A | 2572 | *S. pneumoniae* NZ CP018136 (98.98% ANI) | *S. pneumoniae* NZ LR536837 (98.98% ANI) |
| NS_31 | *S. pneumoniae* | 15A | 13262 | *S. pneumoniae* R6 NC 003098 (98.9% ANI) | *S. pneumoniae* NZ LR216031 (98.82% ANI) |
| NS_32 | *S. pneumoniae* | 19F | 12366 | *S. pneumoniae* A026 NC 022655 (99.6% ANI) | *S. pneumoniae* NZ AP018391 (99.15% ANI) |
| NS_35 | *S. pneumoniae* | 34 | 15478* | *S. pneumoniae* NZ CP019299 (98.78% ANI) | *S. pneumoniae* NZ LR216067 (98.78% ANI) |
| NS_37 | *S. pneumoniae* | 14 | 63 | *S. pneumoniae* G54 NC 011072 (99.64% ANI) | *S. pneumoniae* AP200 NC 014494 (98.86% ANI) |
| NS_41 | *S. pneumoniae* | 19F | 1464 | *S. pneumoniae* A026 NC 022655 (99.9% ANI) | *S. pneumoniae* NZ AP018391 (99.17% ANI) |
| NS_42 | *S. pneumoniae* | 19F | 1464 | *S. pneumoniae* A026 NC 022655 (99.89% ANI) | *S. pneumoniae* NZ AP018391 (99.14% ANI) |
| NS_44 | *S. pneumoniae* | 11A | 6693 | *S. pneumoniae* AP200 NC 014494 (98.85% ANI) | *S. pneumoniae* NZ LR536835 (98.85% ANI) |
| NS_52 | *S. pneumoniae* | 4 | 5120 | *S. pneumoniae* NZ LS483449 (99.07% ANI) | *S. pneumoniae* NZ CP031246 (98.83% ANI) |
| NS_53 | *S. pneumoniae* | 4 | 15484* | *S. pneumoniae* NZ LS483449 (99.04% ANI) | *S. pneumoniae* NZ CP031246 (98.83% ANI) |
| NS_55 | *S. pneumoniae* | 34 | 1439 | *S. pneumoniae* NZ LR216048 (99.49% ANI) | *S. pneumoniae* NZ LR536835 (98.82% ANI) |
| NS_57 | *S. pneumoniae* | 19F | 12366 | *S. pneumoniae* A026 NC 022655 (97.33% ANI) | *S. pneumoniae* NZ LR216048 (97.14% ANI) |
| NS_63 | *S. pneumoniae* | 6E(6B) | 15485* | *S. pneumoniae* NZ LR216021 (98.84% ANI) | *S. pneumoniae* NZ LR216054 (98.77% ANI) |
| NS_64 | *S. pneumoniae* | 6E(6B) | 15485* | *S. pneumoniae* NZ LR216021 (98.8% ANI) | *S. pneumoniae* NZ LR536841 (98.78% ANI) |
| NS_65 | *S. pneumoniae* | 19A | 5108 | *S. pneumoniae* A026 NC 022655 (98.94% ANI) | *S. pneumoniae* NZ LR216035 (98.74% ANI) |
| NS_68 | *S. pneumoniae* | 6E(6B) | 10035 | *S. pneumoniae* NZ LR216021 (98.79% ANI) | *S. pneumoniae* NZ LR536835 (98.79% ANI) |
| NS_71 | *S. pneumoniae* | 19F | 236 | *S. pneumoniae* A026 NC 022655 (99.73% ANI) | *S. pneumoniae* NZ AP018391 (99.28% ANI) |
| NS_72 | *S. pneumoniae* | 6E(6B) | 10035 | *S. pneumoniae* NZ LR216024 (98.78% ANI) | *S. pneumoniae* NZ LR216054 (98.75% ANI) |
| NS_82 | *S. pneumoniae* | untypable | 15486* | *S. pneumoniae* NZ CP018136 (98.7% ANI) | *S. pneumoniae* NZ LR536837 (98.65% ANI) |
| NS_94 | *S. pneumoniae* | 34 | 1439 | *S. pneumoniae* NZ LR216048 (99.56% ANI) | *S. pneumoniae* R6 NC 003098 (98.8% ANI) |
| NS_96 | *S. pneumoniae* | 34 | 1439 | *S. pneumoniae* NZ LR216048 (99.52% ANI) | *S. pneumoniae* NZ LR216016 (98.81% ANI) |
| NS_106 | *S. pneumoniae* | 34 | 1439 | *S. pneumoniae* NZ LR216048 (99.51% ANI) | *S. pneumoniae* NZ LR216016 (98.83% ANI) |
| NS_108 | *S. pneumoniae* | 15B | 1961 | *S. pneumoniae* NZ CP031245 (99.4% ANI) | *S. pneumoniae* NZ LR216058 (98.68% ANI) |
| NS_109 | *S. pneumoniae* | untypable | 15486* | *S. pneumoniae* NZ LR536837 (98.67% ANI) | *S. pneumoniae* NZ CP018136 (98.65% ANI) |
| NS_113 | *S. pneumoniae* | 35B | 8150 | *S. pneumoniae* NZ LR536835 (98.8% ANI) | *S. pneumoniae* NZ LR216037 (98.79% ANI) |
| NS_125 | *S. pneumoniae* | 15B | 1961 | *S. pneumoniae* NZ CP031245 (99.43% ANI) | *S. pneumoniae* NZ LR216058 (98.68% ANI) |
| NS_128 | *S. pneumoniae* | 15C | 15479* | *S. pneumoniae* NZ CP025076 (98.89% ANI) | *S. pneumoniae* NZ CP026549 (98.76% ANI) |
| NS_129 | *S. pneumoniae* | 06A | 15480* | *S. pneumoniae* SPN034183 NC 021028 (98.82% ANI) | *S. pneumoniae* NZ LR216031 (98.8% ANI) |
| NS_130 | *S. pneumoniae* | 15B | 1961 | *S. pneumoniae* NZ CP031245 (99.41% ANI) | *S. pneumoniae* NZ LR216058 (98.69% ANI) |
| NS_135 | *S. pneumoniae* | 15A | 6011 | *S. pneumoniae* NZ LR536835 (99.04% ANI) | *S. pneumoniae* NZ CP019299 (98.93% ANI) |
| NS_143 | *S. pneumoniae* | 6E(6B) | 15481* | *S. pneumoniae* R6 NC 003098 (98.77% ANI) | *S. pneumoniae* NZ CP019299 (98.76% ANI) |
| NS_145 | *S. pneumoniae* | 38 | 8806 | *S. pneumoniae* 70585 NC 012468 (99.12% ANI) | *S. pneumoniae* LR216057 (98.83% ANI) |
| NS_146 | *S. pneumoniae* | 19F | 236 | *S. pneumoniae* A026 NC 022655 (99.72% ANI) | *S. pneumoniae* NZ AP018391 (99.22% ANI) |
| NS_148 | *S. pneumoniae* | 15B | 7768 | *S. pneumoniae* NZ LR216016 (98.74% ANI) | *S. pneumoniae* NZ LR216037 (98.71% ANI) |
| NS_150 | *S. pneumoniae* | 23A | 15482* | *S. pneumoniae* NZ CP018136 (99.04% ANI) | *S. pneumoniae* NZ CP028436 (98.97% ANI) |
| NS_157 | *S. pneumoniae* | 11A | 6693 | *S. pneumoniae* AP200 NC 014494 (98.87% ANI) | *S. pneumoniae* LR536839 (98.83% ANI) |
| NS_158 | *S. pneumoniae* | untypable | 15483* | *S. pneumoniae* NZ LR216048 (98.54% ANI) | *S. pneumoniae* NZ LR216016 (98.47% ANI) |
| NS_159 | *S. pneumoniae* | 06A | 3173 | *S. pneumoniae* NZ CP028436 (99.09% ANI) | *S. pneumoniae* NZ LR536837 (98.87% ANI) |
| NS_166 | *S. pneumoniae* | 23A | 2218 | *S. pneumoniae* NZ CP018136 (99.06% ANI) | *S. pneumoniae* NZ LR536837 (99.0% ANI) |
| NS_169 | *S. pneumoniae* | 06C | 5109 | *S. pneumoniae* NZ LR216021 (98.84% ANI) | *S. pneumoniae* NZ LR216016 (98.84% ANI) |
| NS_171 | *S. pneumoniae* | untypable | 15487* | *S. pneumoniae* NZ CP025076 (98.77% ANI) | *S. pneumoniae* NZ LR216044 (98.61% ANI) |
| NS_174 | *S. pneumoniae* | 35A | 3214 | *S. pneumoniae* NZ CP019299 (99.23% ANI) | *S. pneumoniae* NZ CP031248 (98.84% ANI) |
| NS_182 | *S. pneumoniae* | 6E(6B) | 10035 | *S. pneumoniae* NZ LR216024 (98.78% ANI) | *S. pneumoniae* NZ LR216021 (98.78% ANI) |
| NS_185 | *S. pneumoniae* | untypable | 15487* | *S. pneumoniae* NZ CP025076 (98.76% ANI) | *S. pneumoniae* NZ CP026549 (98.63% ANI) |
| NS_197 | *S. pneumoniae* | 6E(6B) | 10035 | *S. pneumoniae* NZ LR216021 (98.79% ANI) | *S. pneumoniae* NZ LR216054 (98.78% ANI) |
| NS_202 | *S. pneumoniae* | 19F | 4414 | *S. pneumoniae* A026 NC 022655 (99.75% ANI) | *S. pneumoniae* NZ AP018391 (99.24% ANI) |
| NS_207 | *S. pneumoniae* | 11A | 4661 | *S. pneumoniae* NZ CP019299 (99.37% ANI) | *S. pneumoniae* NZ CP031248 (98.89% ANI) |
| NS_238 | *S. pneumoniae* | 35B | 172 | *S. pneumoniae* NZ LR536837 (99.57% ANI) | *S. pneumoniae* NZ CP018136 (99.22% ANI) |
| NS_252 | *S. mitis* | N/A | N/A | *S. mitis* B6 NC 013853 (96.3% ANI) | *S. mitis* NZ CP028415 (94.59% ANI) |
| NS_263 | *S. pneumoniae* | 19F | 4414 | *S. pneumoniae* A026 NC 022655 (99.69% ANI) | *S. pneumoniae* NZ AP018391 (99.18% ANI) |

*These sequence types are identified as novel ones in this study.

**Supplementary Table 4.** MLST profiles of pneumococcal clinical isolates in this study

| Strain | ST | *aroE* | *gdh* | *gki* | *recP* | *spi* | *xpt* | *ddl* |
|---|---|---|---|---|---|---|---|---|
| NS_6 | 855 | 10 | 32 | 19 | 1 | 6 | 14 | 14 |
| NS_9 | 1583 | 15 | 16 | 19 | 15 | 6 | 20 | 71 |
| NS_14 | 7539 | 2 | 11 | 4 | 5 | 6 | 14 | 9 |
| NS_17 | 2697 | 4 | 16 | 19 | 15 | 6 | 20 | 252 |
| NS_19 | 2697 | 4 | 16 | 19 | 15 | 6 | 20 | 252 |
| NS_20 | 855 | 10 | 32 | 19 | 1 | 6 | 14 | 14 |
| NS_21 | 15477* | 2 | 10 | 54 | 1 | 6 | 31 | 6 |
| NS_22 | 10382 | 319 | 13 | 8 | 286 | 25 | 1 | 14 |
| NS_27 | 2572 | 7 | 75 | 9 | 6 | 25 | 6 | 14 |
| NS_28 | 2572 | 7 | 75 | 9 | 6 | 25 | 6 | 14 |
| NS_31 | 13262 | 15 | 5 | 15 | 1 | 15 | 1 | 1 |
| NS_32 | 12366 | 15 | 16 | 19 | 15 | 103 | 20 | 26 |
| NS_35 | 15478* | 10 | 2 | 19 | 16 | 15 | 19 | 14 |
| NS_37 | 63 | 2 | 5 | 36 | 12 | 17 | 21 | 14 |
| NS_41 | 1464 | 4 | 16 | 19 | 15 | 6 | 20 | 106 |
| NS_42 | 1464 | 4 | 16 | 19 | 15 | 6 | 20 | 106 |
| NS_44 | 6693 | 5 | 31 | 8 | 1 | 9 | 1 | 14 |
| NS_52 | 5120 | 162 | 5 | 4 | 15 | 103 | 170 | 5 |
| NS_53 | 15484* | 162 | 5 | 710* | 15 | 103 | 170 | 5 |
| NS_55 | 1439 | 5 | 5 | 6 | 1 | 9 | 14 | 14 |
| NS_57 | 12366 | 15 | 16 | 19 | 15 | 103 | 20 | 26 |
| NS_63 | 15485* | 2 | 5 | 4 | 1 | 6 | 928* | 9 |
| NS_64 | 15485* | 2 | 5 | 4 | 1 | 6 | 928* | 9 |
| NS_65 | 5108 | 15 | 9 | 9 | 1 | 9 | 20 | 26 |
| NS_68 | 10035 | 2 | 5 | 4 | 1 | 6 | 4 | 9 |
| NS_71 | 236 | 15 | 16 | 19 | 15 | 6 | 20 | 26 |
| NS_72 | 10035 | 2 | 5 | 4 | 1 | 6 | 4 | 9 |
| NS_82 | 15486* | 7 | 420 | 4 | 16 | 17 | 12 | 1006* |
| NS_94 | 1439 | 5 | 5 | 6 | 1 | 9 | 14 | 14 |
| NS_96 | 1439 | 5 | 5 | 6 | 1 | 9 | 14 | 14 |
| NS_106 | 1439 | 5 | 5 | 6 | 1 | 9 | 14 | 14 |
| NS_108 | 1961 | 10 | 16 | 128 | 16 | 6 | 14 | 210 |
| NS_109 | 15486* | 7 | 420 | 4 | 16 | 17 | 12 | 1006* |
| NS_113 | 8150 | 10 | 12 | 2 | 1 | 6 | 28 | 14 |
| NS_125 | 1961 | 10 | 16 | 128 | 16 | 6 | 14 | 210 |
| NS_128 | 15479* | 1 | 38 | 9 | 8 | 9 | 3 | 14 |
| NS_129 | 15480* | 164 | 8 | 4 | 10 | 15 | 172 | 14 |
| NS_130 | 1961 | 10 | 16 | 128 | 16 | 6 | 14 | 210 |
| NS_135 | 6011 | 7 | 13 | 4 | 16 | 6 | 1 | 17 |
| NS_143 | 15481* | 2 | 5 | 4 | 1 | 6 | 1 | 8 |
| NS_145 | 8806 | 142 | 13 | 4 | 5 | 17 | 33 | 277 |
| NS_146 | 236 | 15 | 16 | 19 | 15 | 6 | 20 | 26 |
| NS_148 | 7768 | 7 | 187 | 122 | 87 | 17 | 1 | 512 |
| NS_150 | 15482* | 7 | 75 | 9 | 6 | 25 | 50 | 14 |
| NS_157 | 6693 | 5 | 31 | 8 | 1 | 9 | 1 | 14 |
| NS_158 | 15483* | 8 | 8 | 4 | 16 | 16 | 303 | 337 |
| NS_159 | 3173 | 7 | 30 | 8 | 6 | 6 | 6 | 1 |
| NS_166 | 2218 | 7 | 13 | 8 | 6 | 25 | 6 | 14 |
| NS_169 | 5109 | 164 | 8 | 4 | 10 | 15 | 303 | 14 |
| NS_171 | 15487* | 1 | 5 | 711* | 16 | 13 | 1 | 608 |
| NS_174 | 3214 | 10 | 17 | 4 | 38 | 6 | 14 | 6 |
| NS_182 | 10035 | 2 | 5 | 4 | 1 | 6 | 4 | 9 |
| NS_185 | 15487* | 1 | 5 | 711* | 16 | 13 | 1 | 608 |
| NS_197 | 10035 | 2 | 5 | 4 | 1 | 6 | 4 | 9 |
| NS_202 | 4414 | 15 | 16 | 19 | 15 | 6 | 20 | 4 |
| NS_207 | 4661 | 10 | 17 | 4 | 16 | 6 | 1 | 17 |
| NS_238 | 172 | 7 | 13 | 8 | 6 | 25 | 6 | 8 |
| NS_263 | 4414 | 15 | 16 | 19 | 15 | 6 | 20 | 4 |

*These sequence types or loci sequences are identified as novel ones in this study.

## Figure legends

**Supplementary Figure 1** Burden of antimicrobial resistance (AMR) genes in the clinical strains including *Streptococcus mitis*. The reference data was obtained from NCBI Bacterial Antimicrobial Resistance Reference Gene Database. Green, light blue, blue, orange, and gray indicate matches to reference, interrupted, fragmented, partial, and lacked genes, respectively. The clustering tree was generated by ARIBA based on the gene distribution. Graphical data was obtained using Phandango. The genes 23S rRNA, *pbp1a*, *pbp2b*, *pbp2x* and *parC* were identified to contain known variants contributing to antimicrobial resistance.

**Supplementary Figure 2** Burden of genes encoding virulence factors in the clinical strains including *Streptococcus mitis*. The reference data was obtained from virulence factor database (VFDB) full dataset. Green, light blue, blue, orange, and gray indicate matches to reference, interrupted, fragmented, partial, and lacked genes, respectively. The clustering tree was generated using ARIBA based on the gene distribution. Graphical data was obtained using Phandango.

**Supplementary Figure 3** Bayesian phylogenetic relationship of 58 pneumococcal strains isolated in Yangon and 58 public whole genome sequenced strains. The phylogenetic tree was calculated by RAxML using pneumococcal core gene single-nucleotide polymorphisms (SNPs). The tree is midpoint-rooted. The scale bar indicates nucleotide substitutions per site. The color gradation of branches indicates bootstrap value. Inner and outer circles show serotypes and MLSTs, respectively. Serotype and MLST are shown as indicated in the figure. Just one node contains two different serotypes, 19F (Strain 335) and 23F (Strain ATCC 700669).

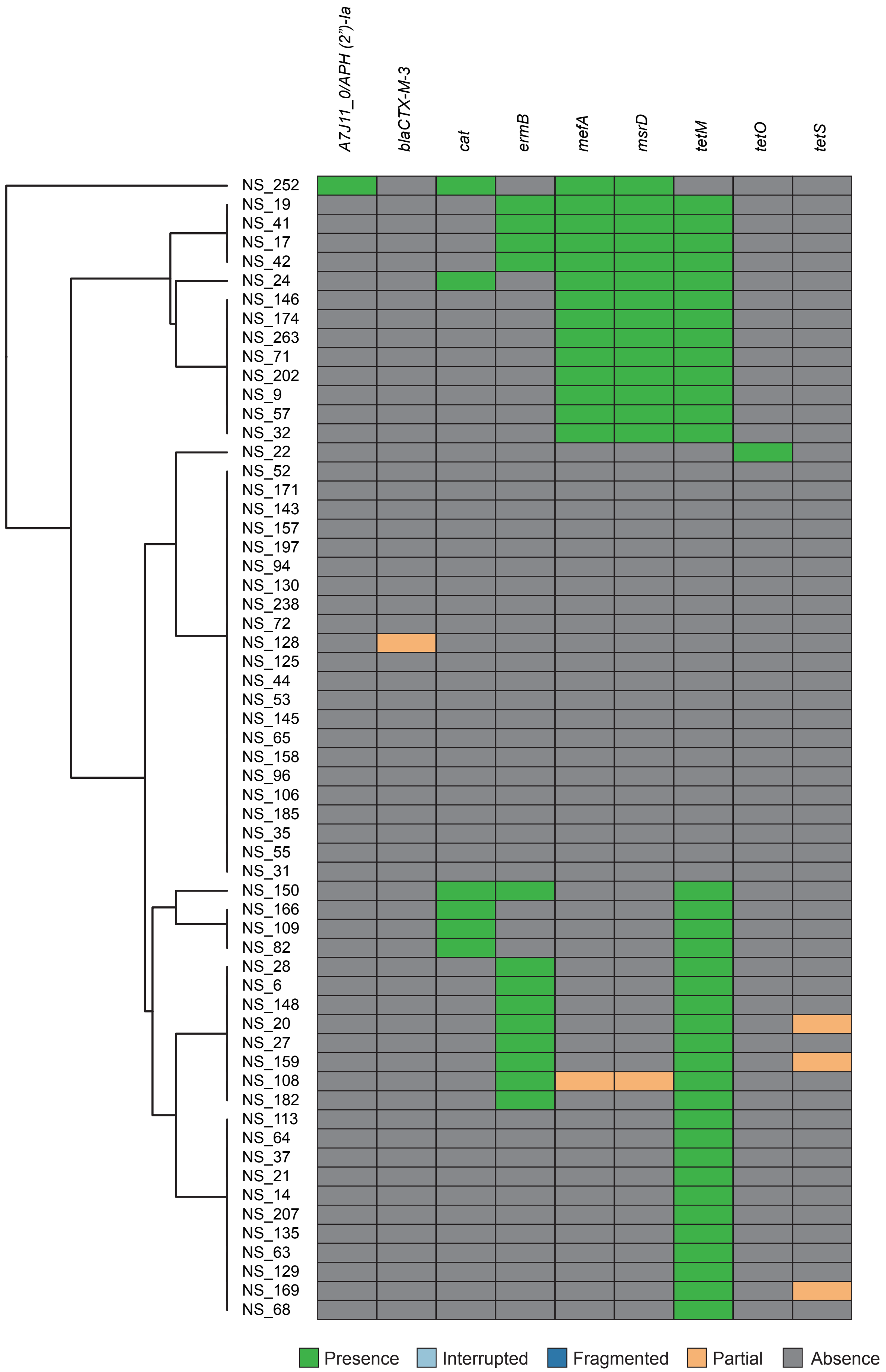


Fig. S1 Yamaguchi et al.

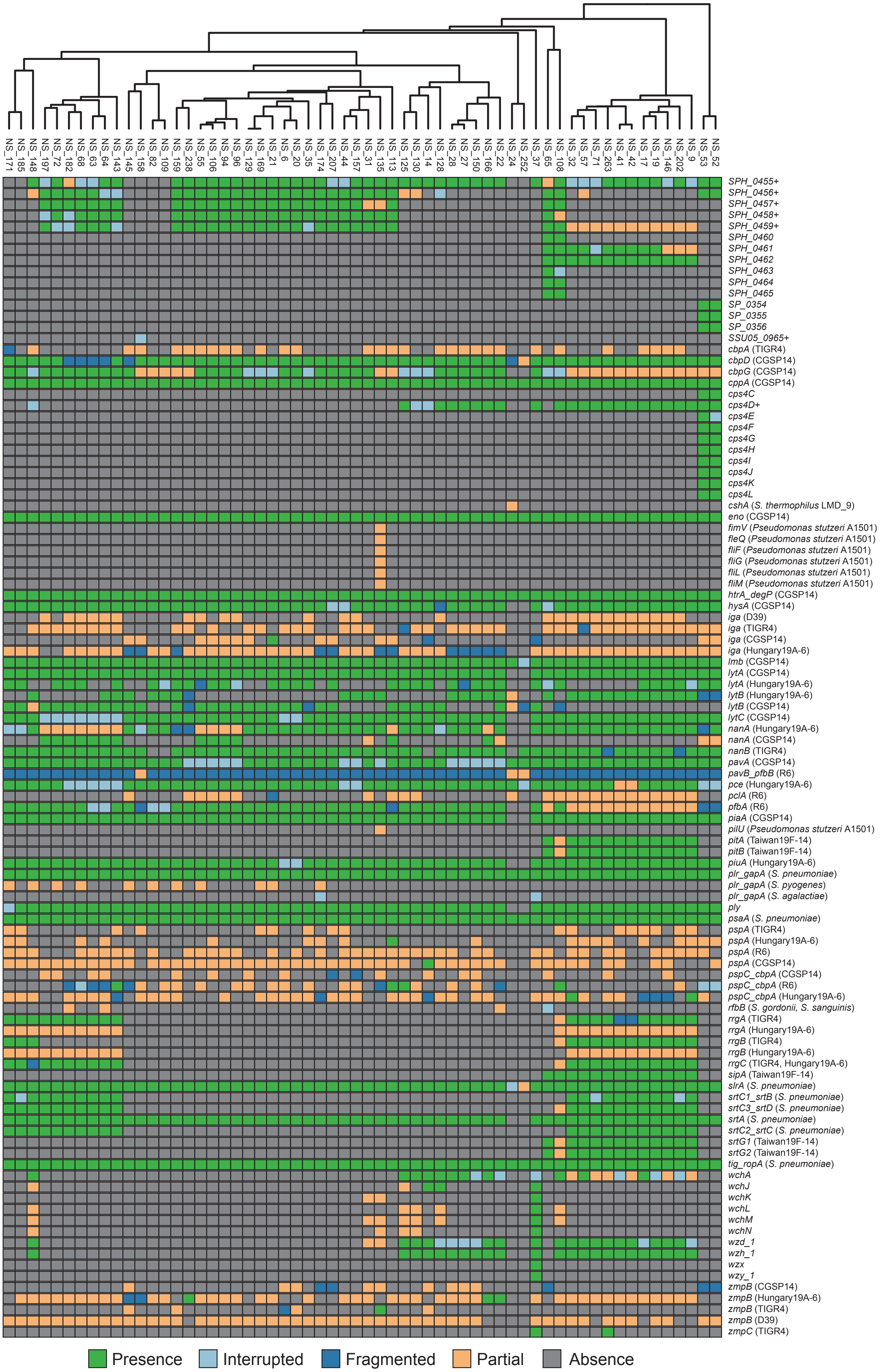


Fig. S2 Yamaguchi et al.

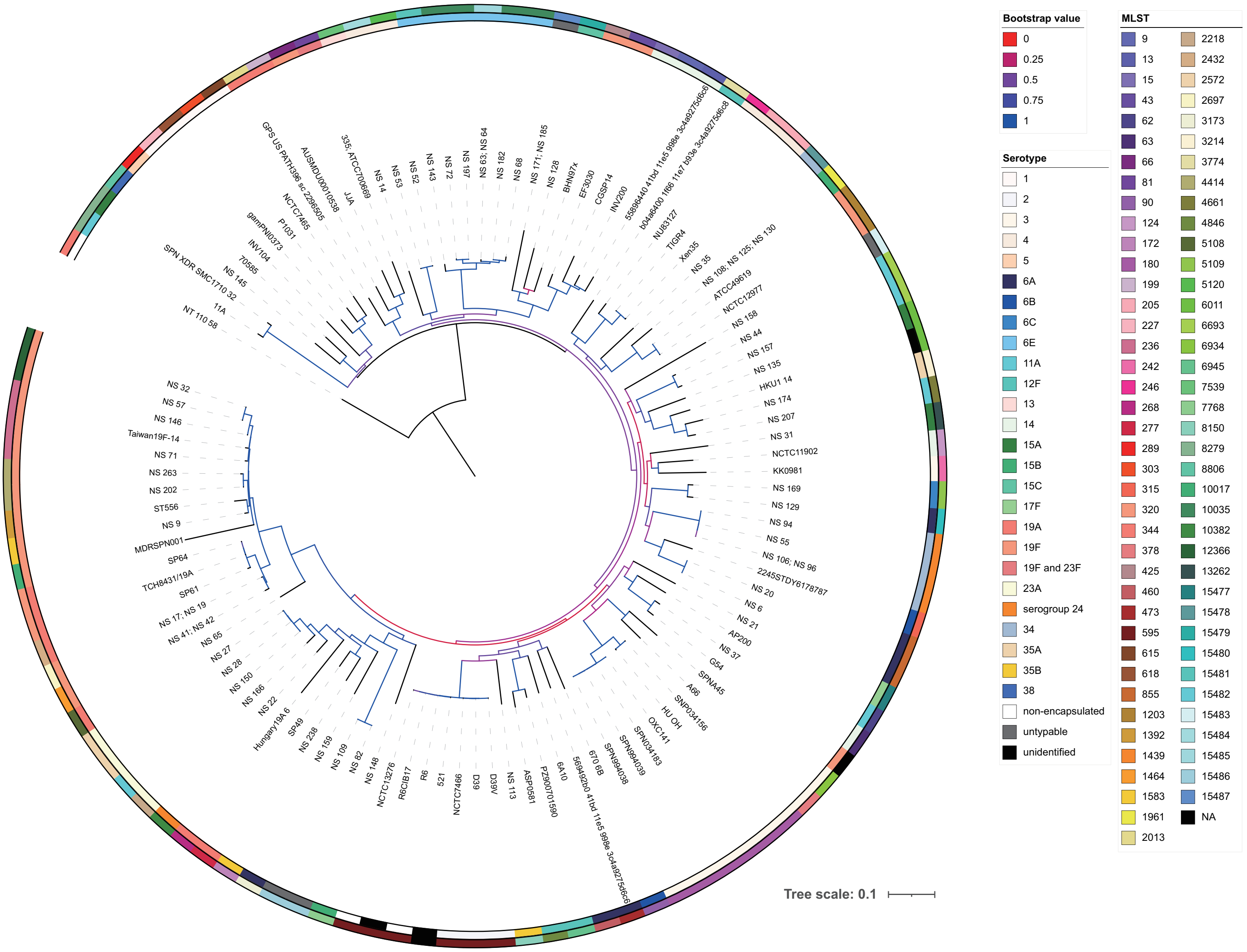


Fig. S3 Yamaguchi et al.
